# Supplementary material for: Genomic Profiling of Highly Aggressive Musculoskeletal Sarcomas Identifies Potential Therapeutic Targets: A Single-Center Experience
Source: Cancers (Basel). 2025 Dec 31;18(1):139. doi: 10.3390/cancers18010139 (PMC12784745; doi:10.3390/cancers18010139)
Supplement: Supplementary file 1 [file cancers-18-00139-s001.zip › cancers-4026395 Table S1.pdf]

VariantPlex Pan Solid Tumor kit panel

ABL1 §	ATR §	BRCA2 §	CHD1 §	EP300 §	FBXW7 §	FOXL2 §	IDH2 §	LZTR1 §	MSH2 §	NOTCH1 §	PIK3CA §#	PTPN11 §	RNF43 §	SMO §	TSC2 §
ACVR1 §	ATRX §	BRIP1 §#	CHEK1 §#	EPCAM §	FGF19 §#	FUBP1 §	JAK1 §	MAP2K1 §	MSH3 §	NOTCH2 §	PIK3CB §#	RAD50 §	ROS1 §	SRC §	TSHR §
AKT1 §#	AURKA §#	CCND1 §#	CHEK2 §#	ERBB2§#	FGFR1 §#	GNA11 §	JAK2 §#	MAP2K2 §	MSH6 §	NOTCH3 §	PIK3R1 §	RAD51 §	SDHA §	SRSF2 §	U2AF1 §
AKT2 §#	B2M §	CCND2 §#	CIC §	ERBB3§#	FGFR2 §#	GNAQ §	JAK3 §	MAP3K1 §	MTOR §	NOTCH4 §	PLCB4 §	RAD51B §#	SDHB §	STAG2 §	VHL §
AKT3 §#	BAP1 §	CCND3 §#	CSF1R §	ERBB4 §	FGFR3 §#	GNAS §	KDM6A §	MDM2 §#	MUC16 §	NPM1 §	PMS2 §	RAD51C §	SDHC §	STK11 §#	XRCC2 §
ALK §#	BARD1 §#	CCNE1 §#	CTNNB1 §	ERCC1 §#	FGFR4 §#	H3F3A §	KDR §#	MDM4 §	MUTYH §	NRAS §#	POLD1 §	RAD51D §	SDHD §	SUFU §	XRCC3 §
APC §	BCOR §	CDH1 §	DAXX §	ERCC2 §#	FH §	H3F3B §	KEAP1 §#	MED12 §	MYC §#	NTRK1 §	POLE §	RAD54L §	SETD2 §	TERT §#	
AR §#	BLM §	CDK12 §#	DDR2 §	ESR1 §#	FLCN §	HIST1H3B §	KIT §#	MEN1 §	MYCN §#	NTRK2 §	PPP2R1A §	RAF1 §#	SF3B1 §	TGFBR2 §	
ARID1A §	BMPR1A §	CDK4 §#	DDX3X §	EZH2 §	FLT1 §	HIST1H3C §	KLF4 §	MET §#	NBN §	NTRK3 §	PPP2R2A §	RB1 §	SMAD2 §	TP53 §	
ARID1B §	BRAF §#	CDK6 §#	DICER1 §	FANCA §	FLT3 §#	HNF1A §	KMT2C §	MLH1 §	NF1 §	PALB2 §	PRKD1 §	RET §#	SMAD4 §	TP63 §	
ARID2 §	BRAF §	CDKN2A §	EGFR §#	FANCI §	FLT4 §	HRAS §	KMT2D §	MPL §	NF2 §	PBRM1 §	PTCH1 §	RHOA §	SMARCA4 §	TRAF7 §	
ATM §#	BRCA1 §	CDKN2B §	EIF1AX §	FANCL §	FOXA1 §	IDH1 §	KRAS §#	MRE11A §	NKX2-1 §	PDGFRA §#	PTEN §#	RICTOR §#	SMARCB1 §	TSC1 §	

§=SNV/delins
# = CNV
