# Supplementary material for: Genomic Profiling of Highly Aggressive Musculoskeletal Sarcomas Identifies Potential Therapeutic Targets: A Single-Center Experience
Source: Cancers (Basel). 2025 Dec 31;18(1):139. doi: 10.3390/cancers18010139 (PMC12784745; doi:10.3390/cancers18010139)
Supplement: Supplementary file 1 [file cancers-18-00139-s001.zip › cancers-4026395-supplementary-Table S2.pdf]

Sample Name	Gene	Variant detected HGVSp	Variant detected HGVSc	AF	ClinVar
**EWS#1**	NOTCH4	NP_004548.3:p.Gly835Val	NM_004557.3:c.2504G>T	0.567	N.A.
	BARD1	NP_000456.2:p.Val507Met	NM_000465.2:c.1518_1519inv	0.449	VCV000140757.26 (Benign/Likely benign)
	MUC16	NP_078966.2:p.Met13472Thr	NM_024690.2:c.40413_40415delinsTAC	0.950	N.A.
	MUC16	NP_078966.2:p.Thr10090Asn	NM_024690.2:c.30269C>A	0.505	N.A.
	PMS2	NP_000526.1:p.Lys541Glu	NM_000535.5:c.1621A>G	0.989	VCV000135065.19 (benign)
	CDKN2A	NP_000068.1:p.Met54IlefsTer66	NM_000077.4:c.161dup	0.900	VCV000819708.5 (uncertain significance, but on rs776792523)
	BRCA2	NP_000050.2:p.Lys3326Ter	NM_000059.3:c.9976A>T	0.502	VCV000038266.67 (benign)
	DAXX	XP_005248915.1:p.Pro654Ser	XM_005248858.1:c.1960C>T	0.496	N.A.
	ATRX	NP_000480.2:p.Thr1582AsnfsTer19	NM_000489.3:c.4744dup	0.806	N.A.
	FLT1	NP_001153392.1:p.Ile548Met	NM_001159920.1:c.1644A>G	0.506	VCV000713357.1 (likely benign)
	RAD51B	NP_001308741.1:p.Arg348Gly	NM_001321812.1:c.1042A>G	0.468	N.A.
	CHEK2	NP_001005735.1:p.Ser548Pro	NM_001005735.1:c.1642T>C	0.496	VCV000141718.13 (uncertain significance)
	MDM4	NP_001191100.1:p.Lys324Gln	NM_001204171.1:c.970A>C	0.513	VCV000424927.12 (uncertain significance)
	MYCN	NP_001280157.1:p.Asp272_Glu275del	NM_001293228.1:c.816_827del	0.455	N.A.
	NOTCH3	NP_000426.2:p.Pro496Leu	NM_000435.2:c.1487C>T	0.527	VCV000256120.14 (likely benign)
**EWS#2**	NOTCH4	NP_004548.3:p.Asp272Gly	NM_004557.3:c.813_815delinsGGG	0.507	N.A.
	AR	NP_000035.2:p.Gln77_Gln80del	NM_000044.3:c.228_239del	0.715	N.A.
	AR	NP_000035.2:p.Gly473dup	NM_000044.3:c.1418_1420dup	0.639	N.A.
	BARD1	NP_000456.2:p.Val507Met	NM_000465.2:c.1518_1519inv	0.533	VCV000140757.26 (Benign/Likely benign)
	MUC16	NP_078966.2:p.Thr5177Ile	NM_024690.2:c.15530C>T	0.513	N.A.
	BMPR1A	NP_004320.2:p.Pro2Thr	NM_004329.2:c.4C>A	0.539	VCV000041782.26 (benign)
	FLT1	NP_002010.2:p.Phe1217del	NM_002019.4:c.3649_3651del	0.520	N.A.
	FLT1	NP_002010.2:p.Glu741Gln	NM_002019.4:c.2221G>C	0.505	N.A.
	CSF1R	NP_001275634.1:p.Ser70Asn	NM_001288705.2:c.209G>A	0.491	N.A.
	PALB2	NP_078951.2:p.Pro864Ser	NM_024675.3:c.2590C>T	0.457	VCV000126669.42 (Benign / Likely Benign)
	RET	NP_065681.1:p.Arg982Cys	NM_020630.4:c.2944C>T	0.500	VCV000013938.31 (conflicting interpretation)
	SDHA	NP_001281261.1:p.Arg31Ter	NM_001294332.1:c.91C>T	0.464	VCV000142601.42 (pathogenic / likely pathogenic)
	TGFBR2	NP_001020018.1:p.Val435Met	NM_001024847.2:c.1303G>A	0.467	N.A.
**EWS#3**	NOTCH4	NP_004548.3:p.Gly942Arg	NM_004557.3:c.2824G>A	0,468	VCV001272247.1 (Benign)
	NOTCH4	NP_004548.3:p.Leu13_Leu16del	NM_004557.3:c.36_47del	0,563	VCV000218641.1 (Benign)
	BARD1	NP_000456.2:p.Val507Met	NM_000465.2:c.1518_1519inv	0.942	VCV000140757.26 (Benign/Likely benign)
	MUC16	NP_078966.2:p.Glu12448Lys	NM_024690.2:c.37341_37342inv	0,481	N.A.
	ROS1		NM_002944.2:c.2598-3C>T	0.477	VCV000789289.4 (Benign)
	PMS2	NP_001308936.1:p.Lys435Glu	NM_001322007.1:c.1303A>G	0.977	VCV000135065.25 (Benign)
	BRCA1	NP_009229.2:p.Met548Ile	NM_007298.3:c.1644G>A	0.476	VCV000041830.84 (Benign)
	KMT2C	NP_733751.2:p.Ile455Met	NM_170606.2:c.1365A>G	0.390	VCV000134736.9 (Benign)
	FLT3	NP_004110.2:p.Asp324Asn	NM_004119.2:c.970G>A	0.558	VCV000134451.1 (Likely benign)
	MUTYH	NP_036354.1:p.Val22Met	NM_012222.2:c.64G>A	0.473	VCV000041760.45 (benign)
	NOTCH2	NP_001186930.1:p.Ala21Thr	NM_001200001.1:c.61G>A	0.449	VCV001240631.8 (Benign)
	ERBB4	NP_001036064.1:p.His374Gln	NM_001042599.1:c.1122T>G	0.554	VCV000770696.11 (Conflicting interpretations of pathogenicity)
	NOTCH1	NP_060087.3:p.Pro1377Ser	NM_017617.3:c.4129C>T	0.400	VCV000134934.28 (Conflicting interpretations of pathogenicity)
	RAD54L	NP_001136020.1:p.Arg738His	NM_001142548.1:c.2213G>A	0.434	N.A.
**EWS#4**	ROS1	NP_002935.2:p.Lys2228_Ser2229delinsGlnCys	NM_002944.2:c.6682_6686delinsCAGTG	0.478	N.A.
	PMS2	NP_001308936.1:p.Lys435Glu	NM_001322007.1:c.1303A>G	0.854	VCV000135065.26 (Benign)
	BMPR1A	NP_004320.2:p.Pro2Thr	NM_004329.2:c.4C>A	0.505	VCV000041782.41 (Benign)
	CDKN2A			CN=0,45	
	FANCA	NP_000126.2:p.Leu1138Val	NM_000135.2:c.3412C>G	0.493	VCV000134265.29 (Benign/Likely benign)
	MSH2	NP_001245210.1:p.Gly256Asp	NM_001258281.1:c.767G>A	0.508	VCV000001762.39 (Benign)
	PTEN		NM_001304717.2:c.154+1del	0.963	VCV000440216.18 (Benign)
	MUTYH	NP_036354.1:p.Ser512Phe	NM_012222.2:c.1535C>T	0.524	VCV000041755.54 (Benign/Likely benign)
	SETD2	NP_054878.5:p.Lys730Glu	NM_014159.6:c.2188A>G	0.419	N.A.
**CDS#1**	MUC16	NP_078966.2:p.Thr4962Ile	NM_024690.2:c.14885C>T	0.481	N.A.
	BRCA1	NP_009225.1:p.Ser1040Asn	NM_007294.3:c.3119G>A	0.467	VCV000017670.122 (benign)
	CCND3	NP_001751.1:p.Ser259Ala	NM_001760.5:c.774_775delinsTG	0.474	N.A.
	FANCA	NP_000126.2:p.Met717Ile	NM_000135.4:c.2151G>T	0.416	VCV000134252.35 (Benign/Likely benign)
	MET	NP_000236.2:p.Thr992Ala	NM_000245.4:c.2974A>G	0.488	VCV000411908.15 (Uncertain significance)
	NOTCH2	NP_077719.2:p.Gly1386Glu	NM_024408.4:c.4157G>A	0.491	N.A.
	CDH1	NP_004351.1:p.Ala592Thr	NM_004360.5:c.1774G>A	0.511	VCV000041783.86 (Benign)
	FOXL2	NP_075555.1:p.Ala179Gly	NM_023067.4:c.536C>G	0.474	VCV000261662.13 (Benign)
	SDHB	NP_002991.2:p.Ser163Pro	NM_003000.3:c.487T>C	0.492	VCV000012792.90 (Conflicting classifications of pathogenicityUncertain significance(3); Benign(18); Likely benign(1))
**CDS#2A**	NOTCH4	NP_004548.3:p.Asp404Val	NM_004557.3:c.1211A>T	0.505	N.A.
	NOTCH4	NP_004548.3:p.Asp272Gly	NM_004557.3:c.813_815delinsGGG	0.495	N.A.
	NOTCH4	NP_004548.3:p.Leu13_Leu16del	NM_004557.3:c.36_47del	0.540	VCV000218641.1 (Benign)
	AR	NP_000035.2:p.Gln77_Gln80del	NM_000044.3:c.228_239del	0.789	VCV000464796.13 (Benign)
	AR	NP_000035.2:p.Gly473dup	NM_000044.3:c.1418_1420dup	0.737	VCV000464786.8 (Benign)
	BARD1	NP_000456.2:p.Gln11His	NM_000465.2:c.33G>T	0.466	VCV000127737.36 (Benign/Likely benign)
	MUC16	NP_078966.2:p.Met13472Thr	NM_024690.2:c.40413_40415delinsTAC	0.978	N.A.
	MUC16	NP_078966.2:p.Ser9207Pro	NM_024690.2:c.27619T>C	0.444	VCV001679859.1 (Likely benign)
	ROS1	NP_002935.2:p.Lys2228_Ser2229delinsGlnCys	NM_002944.2:c.6682_6686delinsCAGTG	0.438	N.A.

	PMS2	NP_001308936.1:p.Lys435Glu	NM_001322007.1:c.1303A>G	0.990	VCV000135065.25 (Benign)	
	SMARCA4	NP_001122317.1:p.Ala321Pro	NM_001128845.1:c.961G>C	0.528	VCV000470471.13 (Conflicting interpretations of pathogenicity)	
	ARID1A	NP_006006.3:p.Thr294Pro	NM_006015.4:c.880A>C	0.605	N.A.	
	AKT1		NM_005163.2:c.567+2T>G	0.613	N.A.	
	BMPR1A	NP_004320.2:p.Pro2Thr	NM_004329.2:c.4C>A	0.526	VCV000041782.41 (Benign)	
	POLD1	NP_001243778.1:p.Arg849His	NM_001256849.1:c.2546G>A	0.472	VCV000220865.35 (Benign/Likely benign)	
	ARID1B	NP_059989.2:p.Pro450dup	NM_017519.2:c.1348_1350dup	0.427	VCV001936461.2 (Likely benign)	
	KDR	NP_002244.1:p.Cys482Arg	NM_002253.2:c.1444T>C	0.487	VCV000012318.5 (Likely benign)	
	KMT2C	NP_733751.2:p.Pro2412Thr	NM_170606.2:c.7234C>A	0.475	VCV000134768.6 (Benign)	
	KMT2D	NP_003473.3:p.Pro2210Leu	NM_003482.3:c.6629C>T	0.526	VCV000094240.30 (Benign/Likely benign)	
	MET	NP_000236.2:p.Ile154Met	NM_000245.2:c.462A>G	0.467	VCV001007762.5 (Uncertain significance)	
	PTEN		NM_001304717.2:c.154+1del	0.961	VCV000440216.18 (Benign)	
	ABL1	NP_009297.2:p.Pro829Leu	NM_007313.2:c.2486C>T	0.496	VCV000133440.7 (Benign)	
	FGF19	NP_005108.1:p.Ser147Thr	NM_005117.2:c.439G>C	0.510	N.A.	
	TERT	NP_001180305.1:p.Gly674Arg	NM_001193376.1:c.2020G>C	0.619	N.A.	Other variants are present but not this specific transversion
**CDS#2B**	NOTCH4	NP_004548.3:p.Asp404Val	NM_004557.3:c.1211A>T	0.519	N.A.	
	NOTCH4	NP_004548.3:p.Asp272Gly	NM_004557.3:c.813_815delinsGGG	0.515	N.A.	
	NOTCH4	NP_004548.3:p.Leu13_Leu16del	NM_004557.3:c.36_47del	0.466	VCV000218641.1 (Benign)	
	AR	NP_000035.2:p.Gln77_Gln80del	NM_000044.3:c.228_239del	0.690	VCV000464796.13 (Benign)	
	AR	NP_000035.2:p.Gly473dup	NM_000044.3:c.1418_1420dup	0.609	VCV000464786.8 (Benign)	
	BARD1	NP_000456.2:p.Gln11His	NM_000465.2:c.33G>T	0.492	VCV000127737.36 (Benign/Likely benign)	
	MUC16	NP_078966.2:p.Met13472Thr	NM_024690.2:c.40413_40415delinsTAC	0.969	N.A.	
	MUC16	NP_078966.2:p.Ser9207Pro	NM_024690.2:c.27619T>C	0.475	VCV001679859.1 (Likely benign)	
	ROS1	NP_002935.2:p.Lys2228_Ser2229delinsGlnCys	NM_002944.2:c.6682_6686delinsCAGTG	0.484	N.A.	
	POLD1	NP_001243778.1:p.Arg849His	NM_001256849.1:c.2546G>A	0.491	VCV000220865.35 (Benign/Likely benign)	
	ARID1B	NP_059989.3:p.Pro533dup	NM_017519.3:c.1597_1599dup	0.400	N.A.	
	KDR	NP_002244.1:p.Cys482Arg	NM_002253.2:c.1444T>C	0.502	VCV000012318.5 (Likely benign)	
	KMT2C	NP_733751.2:p.Pro2412Thr	NM_170606.2:c.7234C>A	0.445	VCV000134768.6 (Benign)	
	KMT2D	NP_003473.3:p.Pro2210Leu	NM_003482.3:c.6629C>T	0.542	VCV000094240.30 (Benign/Likely benign)	
	MET	NP_000236.2:p.Ile154Met	NM_000245.2:c.462A>G	0.486	VCV001007762.5 (Uncertain significance)	
	ABL1	NP_005148.2:p.Pro810Leu	NM_005157.4:c.2429C>T	0.498	VCV000133440.13 (Benign/Likely benign; No data submitted for somatic clinical impact)	
	ERBB3	NP_001973.2:p.Ser1119Cys	NM_001982.3:c.3355A>T	0.453	VCV001178942.2 (Benign; No data submitted for somatic clinical impact)	
	FGF19	NP_005108.1:p.Ser147Thr	NM_005117.2:c.439G>C	0.499	N.A.	
	FGFR3		NM_000142.5:c.445+3A>G	0.982	VCV000255341.16 (Benign; No data submitted for somatic clinical impact)	
**OS#1**	NOTCH4	NP_004548.3:p.Asp272Gly	NM_004557.3:c.813_815delinsGGG	0.974	N.A.	
	PMS2	NP_000526.1:p.Lys541Glu	NM_000535.5:c.1621A>G	0.985	VCV000135065.21, Benign	
	SMARCA4	NP_001122316.1:p.Ala314Pro	NM_001128844.1:c.940G>C	0.465	VCV000581136.8 (This ClinVar record describes a nearly similar alteration (G>A) to what has been found in the patient sample (G>C))	
	ARID1A	NP_006006.3:p.Thr294Pro	NM_006015.4:c.880A>C	0.501	N.A.	
	AKT1		NM_001014431.1:c.566+2T>G	0.480	N.A.	
	BRCA1	NP_009225.1:p.Gln356Arg	NM_007294.3:c.1067A>G	0.463	VCV000041803.67 Benign	
	PDGFRA		NM_001347827.1:c.368-3C>T	0.439	VCV000259954.16 Benign/Likely benign	
	TP53			CN=0,19		
	ATM	NP_000042.3:p.Val410Ala	NM_000051.3:c.1229T>C	0.449	VCV000127332.57 Conflicting interpretations of pathogenicity	
	DAXX	XP_005248915.1:p.Pro654Ser	XM_005248858.1:c.1960C>T	0.487	N.A.	
	SUFU	NP_001171604.1:p.Ala14Pro	NM_001178133.1:c.40G>C	0.526	N.A.	
	ATRX	NP_000480.2:p.Glu723Asp	NM_000489.3:c.2169G>C	0.986	VCV000166714.15 Benign/Likely benign	
	RAD51B	NP_001308738.1:p.Lys243Arg	NM_001321809.1:c.728A>G	0.810	VCV001678952.1, likely benign	
	FGFR4	NP_001278909.1:p.Ser632Pro	NM_001291980.1:c.1894T>C	0.547	N.A.	
	FLCN			CN=4,88		
	MYC			CN=3,25		
	RHOA			CN=2,16		
	SDHD	NP_001263432.1:p.Gly12Ser	NM_001276503.1:c.34G>A	0.501	VCV000006895.43, conflicting interpretation of pathogenicity	
**OS#2A**	BARD1	NP_000456.2:p.Val507Met	NM_000465.2:c.1518_1519inv	0.840	VCV000215471.21 Benign/Likely benign	
	MUC16	NP_078966.2:p.Met13472Thr	NM_024690.2:c.40413_40415delinsTAC	0.980	N.A.	
	MUC16	NP_078966.2:p.Pro11787Leu	NM_024690.2:c.35360C>T	0.788	N.A.	
	ROS1	NP_002935.2: p.Lys2228_Ser2229delinsGlnCys	NM_002944.2: c.6682_6686delinsCAGTG	0.523	N.A.	
	PMS2	NP_000526.1:p.Lys541Glu	NM_000535.5:c.1621A>G	0.986	VCV000135065.21, Benign	
	SMARCA4	NP_001122316.1:p.Ala314Pro	NM_001128844.1:c.940G>C	0.503	VCV000581136.8 (This ClinVar record describes a nearly similar alteration (G>A) to what has been found in the patient sample (G>C))	
	ARID1A	NP_006006.3:p.Thr294Pro	NM_006015.4:c.880A>C	0.540	N.A.	
	AKT1		NM_001014431.1:c.566+2T>G	0.495	N.A.	
	BMPR1A	NP_004320.2:p.Pro2Thr	NM_004329.2:c.4C>A	0.491	VCV000041782.30 Benign	
	BRCA1	NP_009225.1:p.Thr826Lys	NM_007294.3:c.2477C>A	0.407	VCV000037473.46 Benign	
	POLD1	NP_001243778.1:p.Ala599Thr	NM_001256849.1:c.1795G>A	0.623	VCV000220886.15 Conflicting interpretations of pathogenicity	
	ARID1B	NP_001333742.1:p.Gly318_Gly319del	NM_001346813.1:c.939_944del	0.444	N.A.	It is not reported this specific deletion but due to a microsatellite repeat sequence, there are many similar reported.
	ATR	NP_001175.2:p.Val959Met	NM_001184.3:c.2875G>A	0.858	VCV000157974.18 benign	
	CCND3			CN=5,29		
	CDK4			CN=11,85		
	KMT2C	NP_733751.2:p.Leu291Phe	NM_170606.2:c.871C>T	0.830	N.A.	
	TP53	NP_000537.3:p.Arg273His	NM_000546.5:c.818G>A	0.603	VCV000012366.33 Pathogenic , meets criteria to be classified as pathogenic for Li-Fraumeni syndrome.	
	BRIP1	NP_114432.2:p.Val193Ile	NM_032043.2:c.577G>A	0.378	VCV000128192.40 Benign/Likely benign	
	GNAS	NP_001070958.1:p.Pro365Thr	NM_001077490.1:c.1093C>A	0.538	N.A.	
**OS#2B**	BARD1	NP_000456.2:p.Val507Met	NM_000465.2:c.1518_1519inv	0.700	VCV000215471.21 Benign/Likely benign	

	MUC16	NP_078966.2:p.Met13472Thr	NM_024690.2:c.40413_40415delinsTAC	0.978	N.A.
	MUC16	NP_078966.2:p.Pro11787Leu	NM_024690.2:c.35360C>T	0.543	N.A.
	ROS1	NP_002935.2: p.Lys2228_Ser2229delinsGlnCys	NM_002944.2: c.6682_6686delinsCAGTG	0.567	N.A.
	BRCA1	NP_009225.1:p.Thr826Lys	NM_007294.3:c.2477C>A	0.455	VCV000037473.46 Benign
	POLD1	NP_001243778.1:p.Ala599Thr	NM_001256849.1:c.1795G>A	0.503	VCV000220886.15 Conflicting interpretations of pathogenicity
	ATR	NP_001175.2:p.Val959Met	NM_001184.3:c.2875G>A	0.718	VCV000157974.18 benign
	CCND3			CN=3,93	
	CDK4			CN=4,46	
	BRIP1	NP_114432.2:p.Val193Ile	NM_032043.2:c.577G>A	0.443	VCV000128192.40 Benign/Likely benign
**OS#3**	NOTCH4	NP_004548.3:p.Leu16del	NM_004557.3:c.45_47del	0.856	VCV000218642.2 (Benign)
	AR	NP_000035.2:p.Gln80del	NM_000044.3:c.237_239del	0.597	VCV000464798.6 (Benign)
	AR	NP_000035.2:p.Gly473dup	NM_000044.3:c.1418_1420dup	0.621	VCV000464786.8 (Benign)
	MUC16	NP_078966.2:p.Thr4707Ile	NM_024690.2:c.14120C>T	0.564	N.A.
	MUC16			CN=2,13	
	ROS1	NP_002935.2:p.Ile537Met	NM_002944.2:c.1611A>G	0.799	N.A.
	PMS2	NP_000526.1:p.Thr597Ser	NM_000535.5:c.1789A>T	0.480	VCV000041707.37 (Benign)
	SMARCA4	NP_001122316.1:p.Ala321Pro	NM_001128844.1:c.961G>C	0.517	VCV000470471.13 (Conflicting interpretations of pathogenicity)
	AKT1			CN=5,71	
	BMPR1A	NP_004320.2:p.Pro2Thr	NM_004329.2:c.4C>A	0.794	VCV000041782.33 (Benign)
	POLD1	NP_001243778.1:p.Val759Ile	NM_001256849.1:c.2275G>A	0.832	VCV000221038.37 (Conflicting interpretations of pathogenicity)
	ARID1B	NP_001333742.1:p.Ile560Val	NM_001346813.1:c.1678A>G	0.828	VCV000126316.16
	CDKN2A			CN=0,22	
	CDKN2B			CN=0,24	
	FANCA	NP_000126.2:p.Met717Ile	NM_000135.2:c.2151G>T	0.765	VCV000134252.19 (Benign/Likely benign)
	MSH2			CN=3,90	
	EP300	NP_001420.2:p.Gln2223Pro	NM_001429.3:c.6668A>C	0.609	VCV000093744.12 (Benign)
	CIC	NP_001291744.1:p.Arg69Gln	NM_001304815.1:c.206G>A	0.781	N.A.
	DICER1	NP_001182502.1:p.Glu1420del	NM_001195573.1:c.4260_4262del	0.646	N.A.
	EPCAM			CN=4,26	
**OS#4**	NOTCH4	NP_004548.3:p.Asp272Gly	NM_004557.3:c.813_815delinsGGG	0.976	N.A.
	NOTCH4	NP_004548.3:p.Leu16del	NM_004557.3:c.45_47del	0.561	VCV000218642.2 (Benign)
	AR	NP_000035.2:p.Gly473dup	NM_000044.3:c.1418_1420dup	0.620	VCV000464786.8 (Benign)
	SMARCA4	NP_001122316.1:p.Ala321Pro	NM_001128844.1:c.961G>C	0.458	VCV000470471.13 (Conflicting interpretations of pathogenicity)
	ARID1A	NP_006006.3:p.Thr294Pro	NM_006015.4:c.880A>C	0.484	N.A.
	PDGFRA	NP_001334756.1:p.Thr200Ser	NM_001347827.1:c.599C>G	0.566	VCV000041800.14 (Conflicting interpretations of pathogenicity)
	CHD1	NP_001261.2:p.Arg70Gln	NM_001270.2:c.209G>A	0.453	N.A.
	DDR2			CN=2,03	
	MED12	NP_005111.2:p.Ala1904Val	NM_005120.2:c.5711C>T	0.982	VCV000095255.21 (Benign / Likely Benign)
	RB1	NP_000312.2:p.Cys278Ter	NM_000321.2:c.833_834del	0.632	COSV57301262 (the variant reported in this “Genomic Mutation ID” has the same protein effect (termination) but a different genomic sequence effect
	SMAD4		NM_005359.5:c.788-3T>C	0.497	VCV000529906.6 (Uncertain significance)
**OS#5A**	NOTCH4	NP_004548.3:p.Leu16del	NM_004557.3:c.45_47del	0.802	VCV000218642.2 (Benign)
	AR	NP_000035.2:p.Gly470_Gly473dup	NM_000044.3:c.1370_1371insTGGCGGCGGCG(	0.641	VCV001169431.25 (benign)
	ATR	NP_001175.2:p.Ser1142Gly	NM_001184.3:c.3424A>G	0.623	VCV000157977.28 (Conflicting classifications of pathogenicityUncertain significance(8); Likely benign(1))
	PDGFRA		NM_001347830.1:c.407-3C>T	0.632	VCV000259954.30 (Benign/Likely benign)
	TP53	NP_000537.3:p.Val216Met	NM_000546.5:c.646G>A	0.821	VCV000182965.26 (Conflicting interpretations of pathogenicity Pathogenic(4); Likely pathogenic(2); Uncertain significance(1))
	BRCA2	NP_000050.2:p.Thr1915Met	NM_000059.3:c.5744C>T	0.833	VCV000041556.97 (benign)
	RAD50	NP_005723.2:p.Ile94Leu	NM_005732.3:c.280A>C	0.622	VCV000128012.63 (Benign/Likely benign)
	BLM	NP_000048.1:p.Asp64Val	NM_000057.2:c.191A>T	0.717	VCV000127479.41 (Conflicting classifications of pathogenicityUncertain significance(9); Benign(1); Likely benign(2))
**OS#5B**	NOTCH4	NP_004548.3:p.Leu16del	NM_004557.3:c.45_47del	0.857	VCV000218642.2 (Benign)
	AR	NP_000035.2:p.Gln79_Gln80del	NM_000044.6:c.234_239del	0.626	VCV001276845.12 (Benign)
	AR	NP_000035.2:p.Gly470_Gly473dup	NM_000044.6:c.1370_1371insTGGCGGCGGCG(	0.573	VCV001169431.25 (benign)
	BARD1	NP_000456.2:p.Val507Met	NM_000465.4:c.1518_1519inv	0.488	VCV000140757.31 (Benign/Likely benign; No data submitted for somatic clinical impact)
	ATR	NP_001175.2:p.Ser1142Gly	NM_001184.4:c.3424A>G	0.645	VCV000157977.28 (Conflicting classifications of pathogenicityUncertain significance(8); Likely benign(1))
	PDGFRA		NM_006206.6:c.368-3C>T	0.735	VCV000259954.30 (Benign/Likely benign)
	TP53	NP_000537.3:p.Val216Met	NM_000546.6:c.646G>A	0.624	VCV000182965.31 (Pathogenic/Likely pathogenic; No data submitted for somatic clinical impact)
	BRCA2	NP_000050.3:p.Thr1915Met	NM_000059.4:c.5744C>T	0.685	VCV000041556.97 (benign)
	RAD50	NP_005723.2:p.Ile94Leu	NM_005732.4:c.280A>C	0.601	VCV000128012.63 (Benign/Likely benign)
	SUFU	NP_057253.2:p.Ala14Pro	NM_016169.4:c.40G>C	0.401	VCV003963845.1 (Uncertain significance)
	BLM	NP_000048.1:p.Asp64Val	NM_000057.4:c.191A>T	0.689	VCV000127479.41 (Conflicting classifications of pathogenicityUncertain significance(9); Benign(1); Likely benign(2))
	ERBB3	NP_001973.2:p.Ser1119Cys	NM_001982.3:c.3355A>T	0.482	VCV001178942.2 (Benign; No data submitted for somatic clinical impact)
**OS#6A**	NOTCH4	NP_004548.3:p.Asp272Gly	NM_004557.3:c.813_815delinsGGG	0.452	N.A.
	NOTCH4	NP_004548.3:p.Leu13_Leu16del	NM_004557.3:c.36_47del	0.504	VCV000218641.1 (Benign)
	AR	NP_000035.2:p.Gln80del	NM_000044.3:c.237_239del	0.704	VCV000464798.6 (Benign)
	BARD1	NP_000456.2:p.Val507Met	NM_000465.2:c.1518_1519inv	0.637	VCV000140757.26 (Benign/Likely benign)
	ROS1	NP_002935.2:p.Lys2228_Ser2229delinsGlnCys	NM_002944.2:c.6682_6686delinsCAGTG	0.410	N.A.
	PMS2	NP_001308936.1:p.Lys435Glu	NM_001322007.1:c.1303A>G	0.987	VCV000135065.25 (Benign)
	SMARCA4	NP_001122317.1:p.Ala321Pro	NM_001128845.1:c.961G>C	0.455	VCV000470471.13 (Conflicting interpretations of pathogenicity)
	ARID1A	NP_006006.3:p.Thr294Pro	NM_006015.4:c.880A>C	0.510	N.A.
	AKT1		NM_005163.2:c.567+2T>G	0.551	N.A.
	KDR	NP_002244.1:p.Leu31Pro	NM_002253.2:c.92T>C	0.482	N.A.
	ATM	NP_000042.3:p.Pro1354Thr	NM_000051.3:c.4060C>A	0.433	VCV000127379.52 (Conflicting interpretations of pathogenicity Uncertain significance(9); Benign(1); Likely benign(4))

	PTEN		NM_001304717.2:c.154+1del	0.966	VCV000440216.18 (Benign)
	TP63	NP_001316893.1:p.Pro509Thr	NM_001329964.1:c.1525C>A	0.632	VCV000286990.24 (Benign/Likely benign)
**OS#6B**	NOTCH4	NP_004548.3:p.Asp272Gly	NM_004557.4:c.813_815delinsGGG	0.557	N.A.
	NOTCH4	NP_004548.3:p.Leu13_Leu16del	NM_004557.4:c.36_47del	0.576	VCV000218641.1 (Benign)
	AR	NP_000035.2:p.Gln80del	NM_000044.6:c.237_239del	0.625	VCV000464798.6 (Benign)
	BARD1	NP_000456.2:p.Val507Met	NM_000465.4:c.1518_1519inv	0.658	VCV000140757.26 (Benign/Likely benign)
	ROS1	NP_002935.2:p.Lys2228_Ser2229delinsGlnCys	NM_002944.3:c.6682_6686delinsCAGTG	0.416	N.A.
	KDR	NP_002244.1:p.Leu31Pro	NM_002253.3:c.92T>C	0.492	N.A.
	ATM	NP_000042.3:p.Pro1354Thr	NM_000051.4:c.4060C>A	0.407	VCV000127379.52 (Conflicting interpretations of pathogenicity Uncertain significance(9); Benign(1); Likely benign(4))
	TP63	NP_003713.3:p.Pro511Thr	NM_003722.5:c.1531C>A	0.563	VCV000286990.24 (Benign/Likely benign)
**OS#7**	AR	NP_000035.2:p.Gln80dup	NM_000044.6:c.237_239dup	0.437	VCV000434257.43 (Benign/Likely benign)
	SMARCA4	NP_003063.2:p.Ala321Pro	NM_003072.5:c.961G>C	0.408	VCV000470471.16 (Conflicting interpretations of pathogenicity)
	ARID1A	NP_006006.3:p.Thr294Pro	NM_006015.6:c.880A>C	0.450	N.A.
	CCND3	NP_001751.1:p.Ser259Ala	NM_001760.5:c.774_775delinsTG	0.672	N.A.
	CDKN2A	NP_000068.1:p.Ala148Thr	NM_000077.5:c.442G>A	0.572	VCV000041580.40 (Benign)
	DAXX	NP_001341.1:p.Arg230Cys	NM_001350.5:c.688C>T	0.366	N.A.
	KMT2D	NP_003473.3:p.Pro2557Leu	NM_003482.4:c.7670C>T	0.525	VCV000094250.32 (Benign)
	MSH2	NP_000242.1:p.Gly322Asp	NM_000251.3:c.965G>A	0.543	VCV000001762.42 (Benign)
	RAD50	NP_005723.2:p.Val683Ile	NM_005732.4:c.2047G>A	0.479	VCV000185548.36 (Uncertain significance)
	SUFU	NP_057253.2:p.Thr13Pro	NM_016169.4:c.37A>C	0.505	VCV000802631.5 (Conflicting interpretations of pathogenicity)
	EP300	NP_001420.2:p.Pro925Thr	NM_001429.4:c.2773C>A	0.332	VCV000134039.20 (Benign/Likely benign)
	FLT3	NP_004110.2:p.Val194Met	NM_004119.3:c.580G>A	0.648	VCV000134446.5 (Benign)
	APC	NP_000029.2:p.Ala1670Val	NM_000038.6:c.5009C>T	0.473	VCV000135705.56 (Conflicting interpretations of pathogenicity)
	MAP3K1	NP_005912.1:p.Gln237Arg	NM_005921.2:c.710A>G	0.483	VCV000252655.9 (Benign/Likely benign)
	NTRK2	NP_006171.2:p.Ser167Tyr	NM_006180.6:c.500C>A	0.390	VCV001148855.14 (Benign/Likely benign)
**OS#8**	TP53	NP_000537.3:p.Asp281His	NM_000546.6:c.841G>C	0.209	VCV000376588.11 Pathogenic / Likely Pathogenic for germline variant; no data for somatic impact
	SUFU	NP_057253.2:p.Ala14Pro	NM_016169.4:c.40G>C	0.460	N.A.
	CCNE1			CN=4,01	
	BRCA1	NP_009225.1:p.Gln356Arg	NM_007294.4:c.1067A>G	0.561759	VCV000041803.67 Benign
**OS#9**	TP53	NP_000537.3:p.Cys275Tyr	NM_000546.6:c.824G>A	0.415	VCV000215997.46 (Pathogenic/Likely pathogenic on germline variant; oncogenic on somatic impact)
	MUC16	NP_078966.2:p.His6152Gln	NM_024690.2:c.18456C>A	0.847	N.A.
	MUC16	NP_078966.2:p.Thr5177Ile	NM_024690.2:c.15530C>T	0.841	N.A.
	JAK3	NP_000206.2:p.Thr8Met	NM_000215.4:c.23C>T	0.837	VCV000134575.11 (uncertain significance for germline variant, no data for somatic impact)
	CDKN2A			CN=0,26	
	CDKNB			CN=0,27	
	ATRX			CN=0,29	
**OS#10**	TP53	NP_000537.3:p.Asp281His	NM_000546.6:c.841G>C	0.224	VCV000376588.11 (Pathogenic / Likely Pathogenic for germline variant, no data for somatic impact)
	CCNE1			CN=2,56	
	FLCN			CN=3,02	
**OS#11**	ARID1A	NP_006006.3:p.Ala88_Gly93dup	NM_006015.6:c.261_278dup	0.409	VCV000592018.4
	TP53	p.Arg282Trp		0.176	VCV000012364.76 (Pathogenic/Likely Pathogenic for germline variant, Oncogenic for somatic impact)
**OS#12**	ATRX	NP_000480.3:p.Thr668AsnfsTer22	NM_000489.6:c.2003_2021del	0.392	N.A.
	CCND3			CN=2.90	
	CDK4			CN=3.35	
	ERBB3			CN=2.72	
	LZTR1			CN=1.69	
	TRAF7			CN=2.67	
	CDKN2B			CN=0.40	
**OS#13**	SETD2	NP_054878.5:p.Asp1166Tyr	NM_014159.7:c.3496G>T	0.535	N.A.
	RB1			CN=0,31	
**SCOS#1**	SMARCA4	NP_003063.2:p.Ala321Pro	NM_003072.5:c.961G>C	0.551	VCV003320755.1 (Uncertain significance for Germline variant, no data for somatic input)
	SUFU	NP_057253.2:p.Ala14Pro	NM_016169.4:c.40G>C	0.435	N.A.
	TP53	NP_000537.3:p.Glu258Lys	NM_000546.6:c.772G>A	0.153	VCV000012348.31 (Pathogenic / Likely pathogenic for germline variant, no data for somatic impact)
**SEF#1**	SUFU	NP_057253.2:p.Thr13Pro	NM_016169.4:c.37A>C	0.428	VCV000802631.5 Conflicting classifications of pathogenicity for germline variant, no data submitted for somatic impact
	SMARCA4	NP_003063.2:p.Ala314Pro	NM_003072.5:c.940G>C	0.416	VCV002042993.4 Uncertain significance for germline variant; no data for somatic impact
	LZTR1	NP_006758.2:p.Val337Met	NM_006767.4:c.1009G>A	0.689	N.A.
	BRCA2	NP_000050.3:p.Arg2034Cys	NM_000059.4:c.6100C>T	0.603	VCV000041558.123 Benign for germline variant, no data submitted for somatic impact
	MSH3	NP_002430.3:p.Ala61_Pro63dup	NM_002439.5:c.181_189dup	0.365	VCV000638366.3 Uncertain significance for germline variant, no data for somatic impact
	CDKN2A			CN=0,50	
	CDKN2B			CN=0,50	
